# Gastric duplication cyst with elevated CEA level: a case report

**DOI:** 10.1093/jscr/rjy114

**Published:** 2018-05-29

**Authors:** Héloïse Tessely, Aude Montanier, Emmanuel Chasse

**Affiliations:** Department of Abdominal Surgery, Epicura Hospital, 7301 Hornu, Belgium

## Abstract

Gastrointestinal tract duplications are congenital malformations rarely seen in adulthood. Gastric duplications (GD) represent 2–9% of it. Malignant transformation of GD is a rare complication described in the literature. We present the case of a 43-year-old man, who presented an abdominal mass and an elevated CEA level. A total gastrectomy was performed and the histological examination described a gastric duplication cysts (GDC) without malignant transformation. It is not the first case of elevation of CEA in GDC without evidence of malignancy described in the literature. Some authors think that GDC are premalignant lesions that envolve with the time to carcinomas. It is recommend that once the GDC is diagnosed to remove surgically the entire cyst even if the patient is asymptomatic.

## INTRODUCTION

Gastrointestinal tract duplications are rare congenital malformations with a reported incidence of ~1 in 4500 live births (1).

The definition of gastrointestinal tract duplication is a tubular structure that is adjacent to, a part of the gastrointestinal tract, that can be situated from the tongue to the anus. Gastrointestinal tract duplications share a common muscular wall and blood supply but have a separate mucosal lining (2, 3).

In descending order of frequency, they can be found in the ileum (33%), the oesophagus (20%), the jejunum (10%), the colon (13%) and in the stomach. Gastric duplication cysts (GDC) are only 2–9% of all the gastrointestinal duplications (3).

The great majority of cases of gastric duplication (GD) are diagnosed in the early childhood, before the age of 12, with a majority in the first year of life (4).

GD can communicate with the gastric lumen but, in most of the cases (80%) they are non-communicating (1). The most common location is the distal greater curvature followed by the posterior wall, the lesser curvature the anterior wall and pylorus.

In up to 50% of the cases, GD are associated with other malformations mainly located in the oesophagus, the vertebrae and the pancreas (4).

Symptoms due to GD can be abdominal pain, abdominal distension due to the mass, nausea, vomiting, dysphagia, dyspepsia, weight loss or can be asymptomatic in most of the cases in adults. Some complications such as haemorrhage, perforation, malignant transformation and gastric obstruction can occur but are extremely rare (2).

Although it is difficult to diagnose GDC preoperatively, some imaging techniques can help us in the differential diagnosis. Actually, the most recommended are EUS and MRI. EUS can help us to characterize the lesion and MRI can give us a morphological exploration. The definitive diagnose of GDC can only be made by surgical removal and histopathological examinations (5).

There is no worldwide accepted treatment but the literature advises total resection of the GDC to prevent complications from it such as malignant transformation (5).

## CASE REPORT

A 43-year-old man with a history of hypertension, hyperuricemia, android obesity and bilateral carpal tunnel, presented at his general practitioner with intermittent pain in right lumbar region.

On abdominal examination we palpates an epigastric mass, the rest of the physical examination was normal.

A abdominal CT scan was realized and demonstrates the presence of a voluminous mass of 24 × 15 × 13 cm^3^, partially cystic, located in the epigastric region, between the left lobe of the liver, the stomach, the pancreas and the transverse colon. The differential diagnoses are: cystic lymphangioma, cystic mesothelioma, non-communicating GD and cystic GIST.

A biology carried out at the same time an increased CEA level of 281 µg/L, and CA 19-9 being within normal limits.

**Figure 1:**
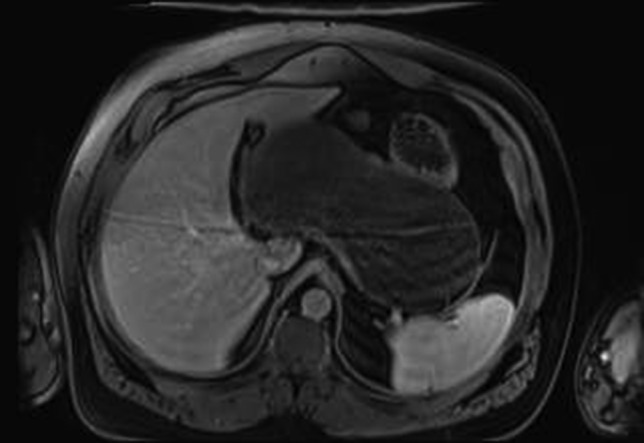
MRI imaging showing a cystic mass located between gastric antrum, pancreas, spleen and hepatic hilum.

MRI gave a diagnosis of a non-communicating GD (Fig. 1). The PETCT showed a moderately hypermetabolic uptake in the posterior wall of the mass, which could not differentiate a benign or malignant origin. Gastric endoscopy showed an extrinsic compression of the gastric body, with a normal mucosa.

**Figure 2:**
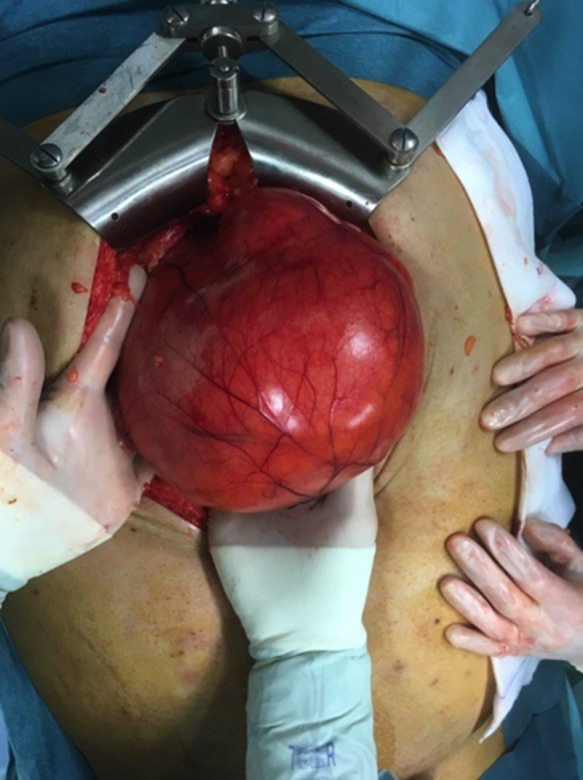
GDC full of mucus.

A decision for laparotomy surgery was made in view of the possible neoplastic degeneration and the difficulties caused by the volume of the mass. During the surgery, a GD of 30 cm of major axis, located along the entirety of the small curvature and under tension was highlighted (Fig. 2). The incision of the cyst allowed 2 L of mucus to be evacueted. The lumen of the cyst did not communicate with that of the stomach. We performed a total gastrectomy with a roux-en-y loop oesophago-jejunostomy and a lymphadenectomy.

**Figure 3:**
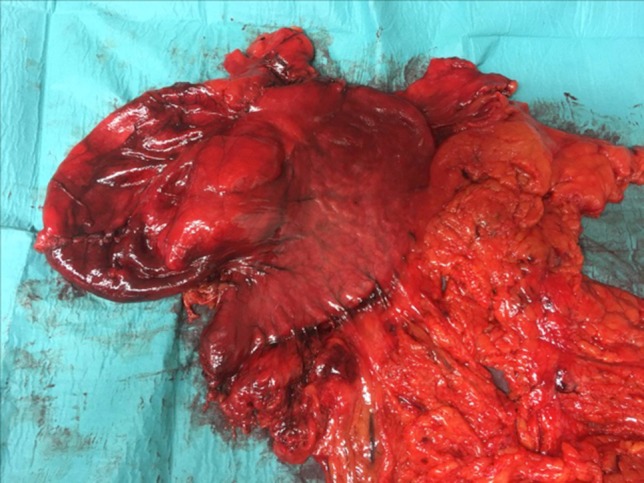
GDC Surgical piece:the GDC attached to the stomach and the omentum.

The histological examination described a normal stomach with a GDC without communication and adjacent to the little curvate. The size of the empty GDC was 21 cm × 10 × 9.5 cm (Fig. 3). The sample of the GDC showed a mucus-secreting epithelium with some zones of ulceration. The wall was thick and had a fibrous and muscular aspect.

No evidence of dysplasia or metaplasia was found in the stomach and neither in the GDC.

The immune marking of pancytokeratine was normal and the KI67 proliferation was little elevated. The PAS coloration showed mucus but there was no signet ring cell. Overall, 18 sampled lymph nodes were free of malignancy.

The diagnosis was a total GDC with no malignant transformation.

The postoperative course was uneventful and, the patient was discharged on the 10-day postoperatively. At 6 months postoperatively the patient is currently well. The CEA is currently at 3,06 mg/L.

## DISCUSSION

The criteria of the definition of GDC are: (i) the wall of the cyst is contiguous with those of the stomach; (ii) the cyst is surrounded by smooth muscle that is continuous of those of the stomach and (iii) the cyst wall is lined by epithelium of gastric or any other type of gut mucosa (6).

Although it is difficult to diagnose GDC preoperatively, some imaging techniques can help in the differential diagnosis. Even if there is no worldwide accepted diagnostic algorithm, most recommended complementary exams are EUS and MRI.

EUS has the advantage of showing the nature of the mass whether solid or cystic and to the borders of the lesion and it useful to do a cytopunction to exclude malignant tissue (5, 6).

MRI could help making a preoperative diagnosis. But the definitive diagnose of GDC can only be made by surgical removal and histological examinations (5).

Malignant transformation of GDC is extremely rare, only 11 cases have been reported in the English-language literature to the date, summarized in Table 1 (7).

**Table 1:** Clinicopathological data in cases of malignant transformation of gastric duplication cysts.

Reference	Age/sex	Symptoms	Histology	Size	CEA/CA 19-9
Mayo *et al.*	64/F	Weakness, anorexia	ADC	6 cm	NA
Coit and Mies	50/M	Vomiting	Epithelial malignancy	17 cm	NA
	72/F	Abdominal pain	ADC	3.2 cm	NA
Kuraoka *et al.*	40/M	Fever, back pain	ADC	7 cm	NA
	71/F	Abdominal pain, anorexia	ADC	8 cm	NA
	56/M	Vomiting, weight loss	ADC	10 cm	NA
Home *et al.*	40/M	Abdominal pain	NEC	12 cm	NA
Barussaud *et al.*	67/F	Abdominal mass, weight loss	Mixed ADC and SCC	NA	CA 19-9 ↑
Zheng *et al.*	25/M	Asymptomatic	ADC	8 cm	CEA ↑
Yamasaki *et al.*	47/F	Asymptomatic	ADC	10 cm	CA 19-9 ↑

F, female; M, male; ADC: adenocarcinoma; SCC, squamous cell carcinoma; NA, non available.

Most of the cases are adenocarcinomas (8/11), other histologic types have been described such as neuroendocrine carcinoma, squamous cell carcinoma and epithelial carcinoma. Most patients are symptomatic and only two cases of malignant transformation were asymptomatic (7).

In addition to our case report, tumour markers were available in only three case reports. CEA was elevated in 1 (Zheng *et al.*), and CA 19-9 in 2 (Barussaud *et al.* and Yamasaki *et al.*).

In our case the CEA was elevated without any carcinoma to be found. It seems that it is not the first case of elevation of CEA and/or CA 19-9 without evidence of malignancy.

Ikehata *et al.* reported a case of a young man with a GDC with markedly elevated CA 19-9 and Tatsuya *et al.* an ileal duplication cyst with elevated CEA and CA 19-9 without evidence of malignancy (8). This could indicate that gastrointestinal tract duplications are premalignant lesions that evolve with the time to carcinomas, and because removed early after diagnosed in children explain that malignant lesions are only found in adults (9).

This theory of premalignant lesion could explain the elevation of CA 19-9 and CEA before any cancer is found. This is why some authors recommend that once the GDC is diagnosed to remove surgically the entire cyst even if the patient is asymptomatic (10).

So, the gold standard treatment of GDC is the total excision of the cyst (2).

For patients who can tolerate surgery we think that the treatment should be the surgical removing of the GDC, no matters if he is symptomatic or not.

Many techniques are described such as excision of the common wall, bypass and partial or total gastrectomy. When a malignant transformation is suspected total gastrectomy with lymphadenectomy should be performed (5).

Laparotomy or laparoscopic approaches are both possible, in the literature six cases of GDC where already treated by minimal invasive techniques (5).

The choice of the technique is based on characteristics such as the diameter of the GDC, prior abdominal surgery, possible malignant transformation and surgical experience of the surgeon. In our case we choose to do an open surgery because of diameter of the cyst (25 cm) and the possible malignant transformation.

## CONCLUSION

GDC is a rare entity and even more in adults. GDC are difficult to diagnose preoperatively, some techniques such as MRI and EUS can help in the differential diagnosis.

Malignant transformation is a rare complication of GDC. We suspect that GDC are premalignant lesions that must be removed even if patients are asymptomatic.

CEA level may be elevated in the absence of malignancy.

The treatment must be surgical and the definitive diagnosis is on pathologic examination of resected surgical piece.
